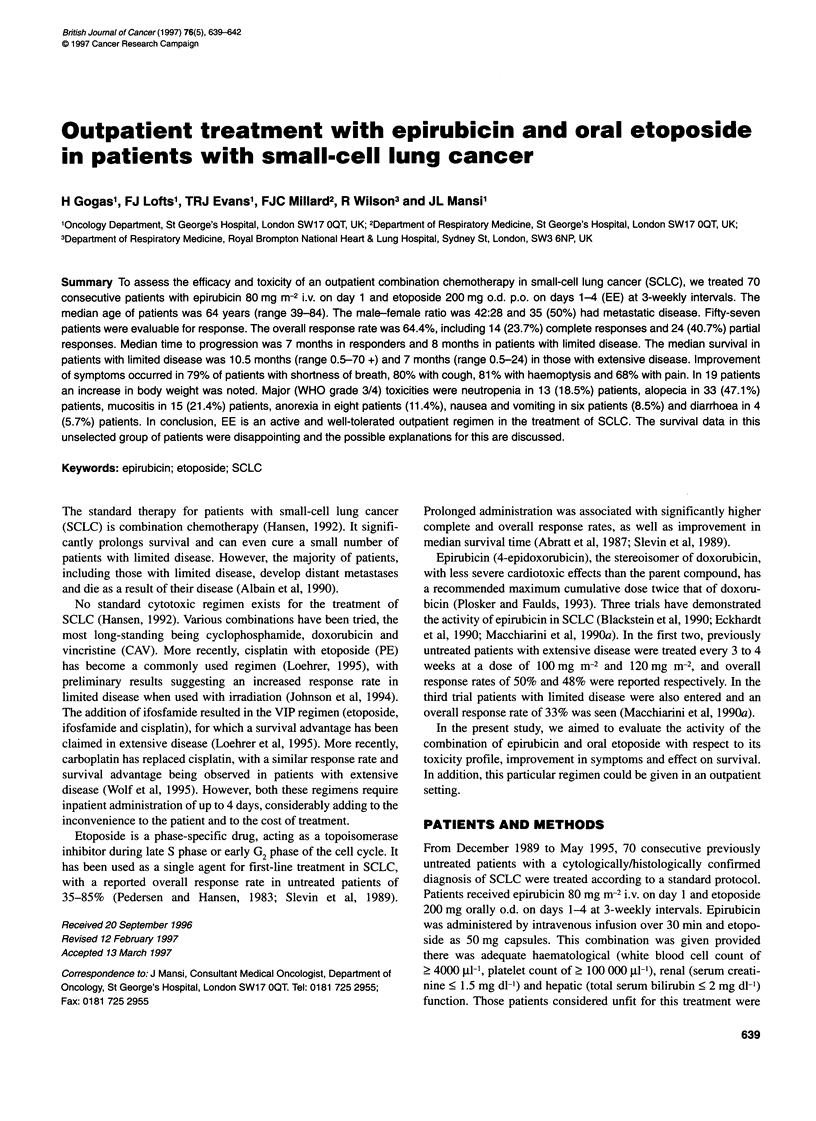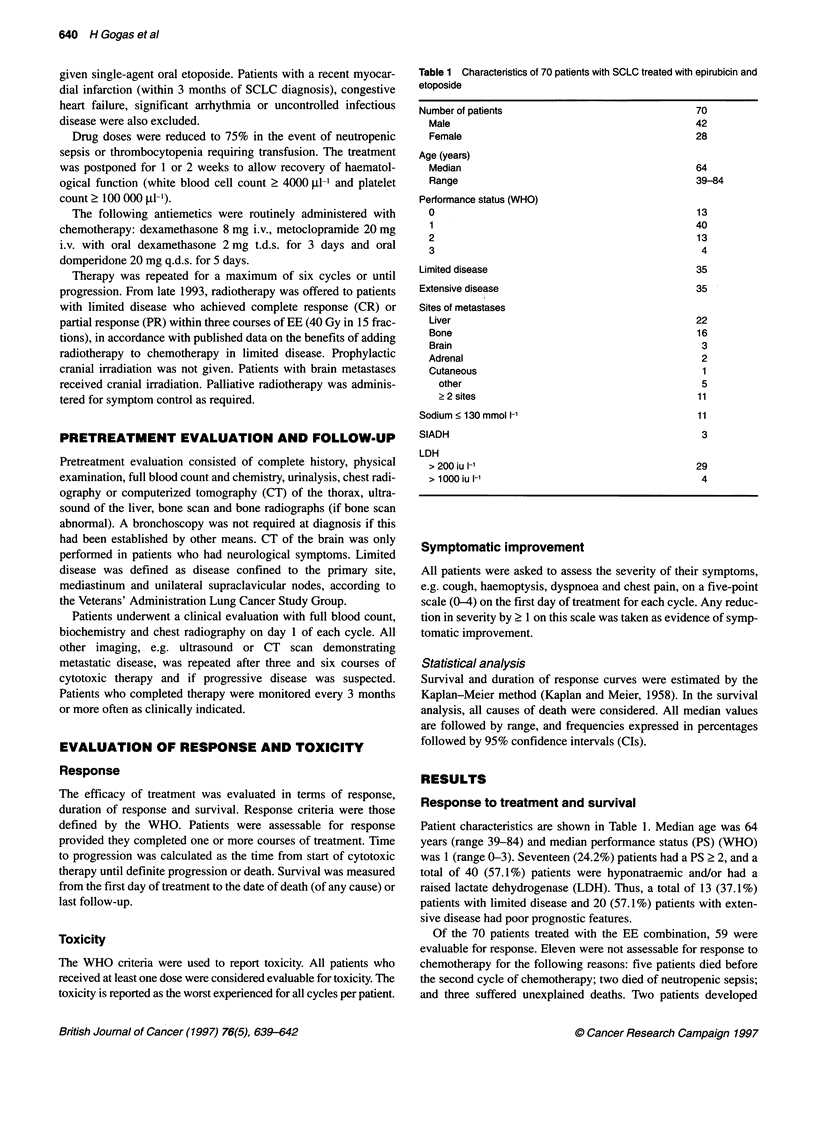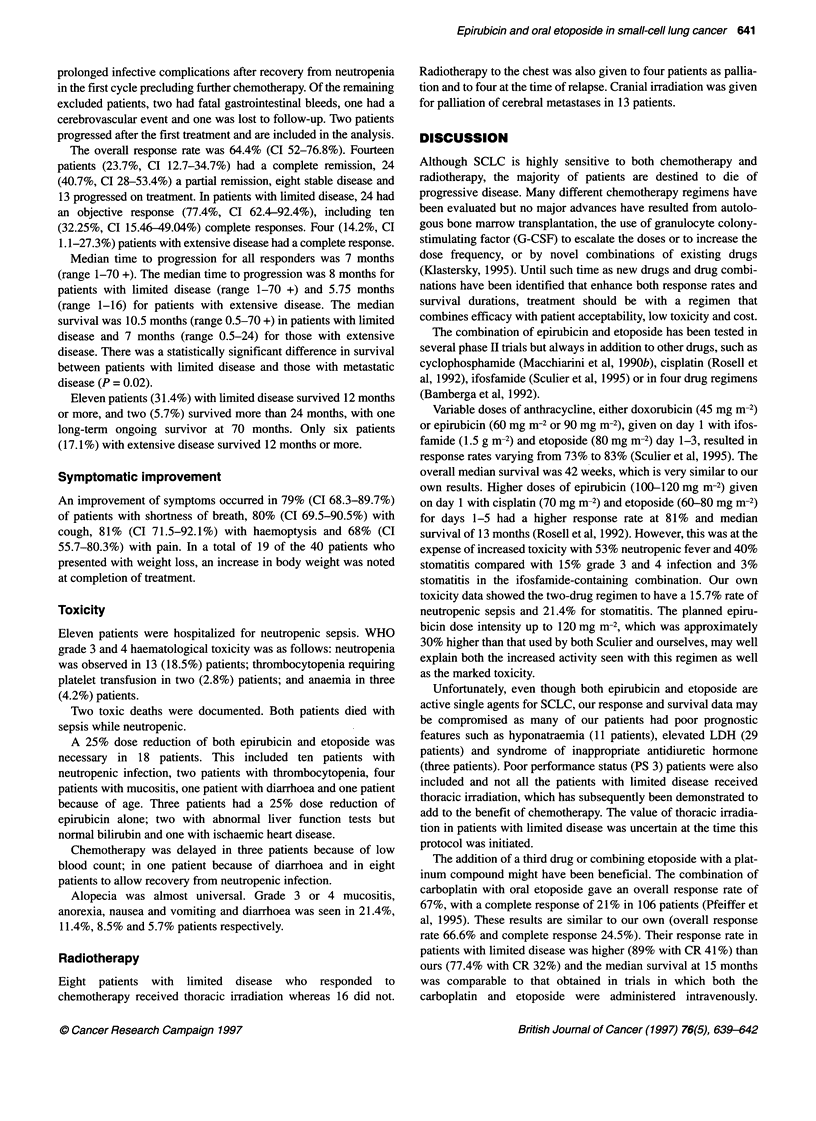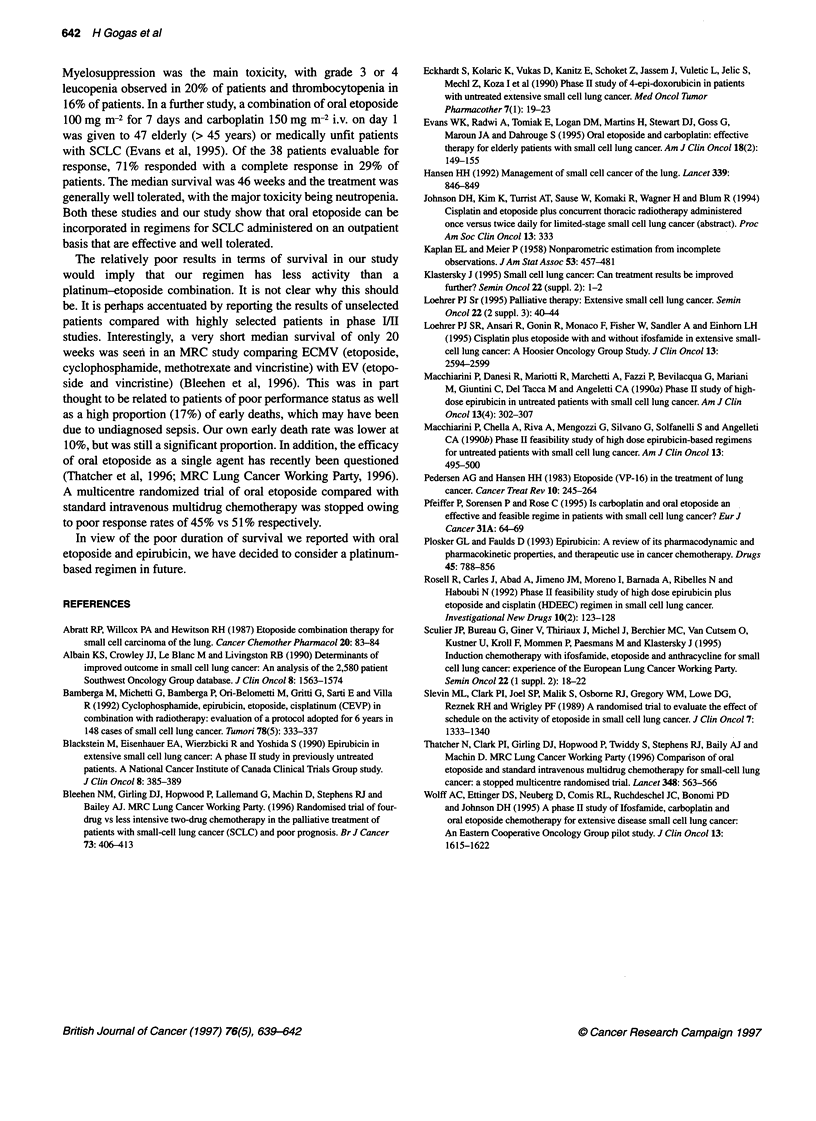# Outpatient treatment with epirubicin and oral etoposide in patients with small-cell lung cancer.

**DOI:** 10.1038/bjc.1997.438

**Published:** 1997

**Authors:** H. Gogas, F. J. Lofts, T. R. Evans, F. J. Millard, R. Wilson, J. L. Mansi

**Affiliations:** Oncology Department, St George's Hospital, London, UK.

## Abstract

To assess the efficacy and toxicity of an outpatient combination chemotherapy in small-cell lung cancer (SCLC), we treated 70 consecutive patients with epirubicin 80 mg m(-2) i.v. on day 1 and etoposide 200 mg o.d. p.o. on days 1-4 (EE) at 3-weekly intervals. The median age of patients was 64 years (range 39-84). The male-female ratio was 42:28 and 35 (50%) had metastatic disease. Fifty-seven patients were evaluable for response. The overall response rate was 64.4%, including 14 (23.7%) complete responses and 24 (40.7%) partial responses. Median time to progression was 7 months in responders and 8 months in patients with limited disease. The median survival in patients with limited disease was 10.5 months (range 0.5-70 +) and 7 months (range 0.5-24) in those with extensive disease. Improvement of symptoms occurred in 79% of patients with shortness of breath, 80% with cough, 81% with haemoptysis and 68% with pain. In 19 patients an increase in body weight was noted. Major (WHO grade 3/4) toxicities were neutropenia in 13 (18.5%) patients, alopecia in 33 (47.1%) patients, mucositis in 15 (21.4%) patients, anorexia in eight patients (11.4%), nausea and vomiting in six patients (8.5%) and diarrhoea in 4 (5.7%) patients. In conclusion, EE is an active and well-tolerated outpatient regimen in the treatment of SCLC. The survival data in this unselected group of patients were disappointing and the possible explanations for this are discussed.


British Journal of Cancer (1997) 76(5), 639–642
© 1997 Cancer Research Campaign

### Outpatient treatment with epirubicin and oral etoposide in patients with small-cell lung cancer

**H Gogas[1], FJ Lofts[1], TRJ Evans[1], FJC Millard[2], R Wilson[3] and JL Mansi[1]**

[1]Oncology Department, St George's Hospital, London SW17 0QT, UK; [2]Department of Respiratory Medicine, St George's Hospital, London SW17 0QT, UK; [3]Department of Respiratory Medicine, Royal Brompton National Heart & Lung Hospital, Sydney St, London, SW3 6NP, UK

**Summary** To assess the efficacy and toxicity of an outpatient combination chemotherapy in small-cell lung cancer (SCLC), we treated 70 consecutive patients with epirubicin 80 mg $m^{-2}$ i.v. on day 1 and etoposide 200 mg o.d. p.o. on days 1–4 (EE) at 3-weekly intervals. The median age of patients was 64 years (range 39–84). The male–female ratio was 42:28 and 35 (50%) had metastatic disease. Fifty-seven patients were evaluable for response. The overall response rate was 64.4%, including 14 (23.7%) complete responses and 24 (40.7%) partial responses. Median time to progression was 7 months in responders and 8 months in patients with limited disease. The median survival in patients with limited disease was 10.5 months (range 0.5–70 +) and 7 months (range 0.5–24) in those with extensive disease. Improvement of symptoms occurred in 79% of patients with shortness of breath, 80% with cough, 81% with haemoptysis and 68% with pain. In 19 patients an increase in body weight was noted. Major (WHO grade 3/4) toxicities were neutropenia in 13 (18.5%) patients, alopecia in 33 (47.1%) patients, mucositis in 15 (21.4%) patients, anorexia in eight patients (11.4%), nausea and vomiting in six patients (8.5%) and diarrhoea in 4 (5.7%) patients. In conclusion, EE is an active and well-tolerated outpatient regimen in the treatment of SCLC. The survival data in this unselected group of patients were disappointing and the possible explanations for this are discussed.

**Keywords:** epirubicin; etoposide; SCLC

The standard therapy for patients with small-cell lung cancer (SCLC) is combination chemotherapy (Hansen, 1992). It significantly prolongs survival and can even cure a small number of patients with limited disease. However, the majority of patients, including those with limited disease, develop distant metastases and die as a result of their disease (Albain et al, 1990).

No standard cytotoxic regimen exists for the treatment of SCLC (Hansen, 1992). Various combinations have been tried, the most long-standing being cyclophosphamide, doxorubicin and vincristine (CAV). More recently, cisplatin with etoposide (PE) has become a commonly used regimen (Loehrer, 1995), with preliminary results suggesting an increased response rate in limited disease when used with irradiation (Johnson et al, 1994). The addition of ifosfamide resulted in the VIP regimen (etoposide, ifosfamide and cisplatin), for which a survival advantage has been claimed in extensive disease (Loehrer et al, 1995). More recently, carboplatin has replaced cisplatin, with a similar response rate and survival advantage being observed in patients with extensive disease (Wolf et al, 1995). However, both these regimens require inpatient administration of up to 4 days, considerably adding to the inconvenience to the patient and to the cost of treatment.

Etoposide is a phase-specific drug, acting as a topoisomerase inhibitor during late S phase or early $G_2$ phase of the cell cycle. It has been used as a single agent for first-line treatment in SCLC, with a reported overall response rate in untreated patients of 35–85% (Pedersen and Hansen, 1983; Slevin et al, 1989). Prolonged administration was associated with significantly higher complete and overall response rates, as well as improvement in median survival time (Abratt et al, 1987; Slevin et al, 1989).

Epirubicin (4-epidoxorubicin), the stereoisomer of doxorubicin, with less severe cardiotoxic effects than the parent compound, has a recommended maximum cumulative dose twice that of doxorubicin (Plosker and Faulds, 1993). Three trials have demonstrated the activity of epirubicin in SCLC (Blackstein et al, 1990; Eckhardt et al, 1990; Macchiarini et al, 1990*a*). In the first two, previously untreated patients with extensive disease were treated every 3 to 4 weeks at a dose of 100 mg $m^{-2}$ and 120 mg $m^{-2}$, and overall response rates of 50% and 48% were reported respectively. In the third trial patients with limited disease were also entered and an overall response rate of 33% was seen (Macchiarini et al, 1990*a*).

In the present study, we aimed to evaluate the activity of the combination of epirubicin and oral etoposide with respect to its toxicity profile, improvement in symptoms and effect on survival. In addition, this particular regimen could be given in an outpatient setting.

### PATIENTS AND METHODS

From December 1989 to May 1995, 70 consecutive previously untreated patients with a cytologically/histologically confirmed diagnosis of SCLC were treated according to a standard protocol. Patients received epirubicin 80 mg $m^{-2}$ i.v. on day 1 and etoposide 200 mg orally o.d. on days 1–4 at 3-weekly intervals. Epirubicin was administered by intravenous infusion over 30 min and etoposide as 50 mg capsules. This combination was given provided there was adequate haematological (white blood cell count of $\geq$ 4000 $\mu l^{-1}$, platelet count of $\geq$ 100 000 $\mu l^{-1}$), renal (serum creatinine $\leq$ 1.5 mg $dl^{-1}$) and hepatic (total serum bilirubin $\leq$ 2 mg $dl^{-1}$) function. Those patients considered unfit for this treatment were

*Received 20 September 1996*
*Revised 12 February 1997*
*Accepted 13 March 1997*

*Correspondence to:* J Mansi, Consultant Medical Oncologist, Department of Oncology, St George's Hospital, London SW17 0QT. Tel: 0181 725 2955; Fax: 0181 725 2955

given single-agent oral etoposide. Patients with a recent myocardial infarction (within 3 months of SCLC diagnosis), congestive heart failure, significant arrhythmia or uncontrolled infectious disease were also excluded.

Drug doses were reduced to 75% in the event of neutropenic sepsis or thrombocytopenia requiring transfusion. The treatment was postponed for 1 or 2 weeks to allow recovery of haematological function (white blood cell count ≥ 4000 $\mu l^{-1}$ and platelet count ≥ 100 000 $\mu l^{-1}$).

The following antiemetics were routinely administered with chemotherapy: dexamethasone 8 mg i.v., metoclopramide 20 mg i.v. with oral dexamethasone 2 mg t.d.s. for 3 days and oral domperidone 20 mg q.d.s. for 5 days.

Therapy was repeated for a maximum of six cycles or until progression. From late 1993, radiotherapy was offered to patients with limited disease who achieved complete response (CR) or partial response (PR) within three courses of EE (40 Gy in 15 fractions), in accordance with published data on the benefits of adding radiotherapy to chemotherapy in limited disease. Prophylactic cranial irradiation was not given. Patients with brain metastases received cranial irradiation. Palliative radiotherapy was administered for symptom control as required.

### PRETREATMENT EVALUATION AND FOLLOW-UP

Pretreatment evaluation consisted of complete history, physical examination, full blood count and chemistry, urinalysis, chest radiography or computerized tomography (CT) of the thorax, ultrasound of the liver, bone scan and bone radiographs (if bone scan abnormal). A bronchoscopy was not required at diagnosis if this had been established by other means. CT of the brain was only performed in patients who had neurological symptoms. Limited disease was defined as disease confined to the primary site, mediastinum and unilateral supraclavicular nodes, according to the Veterans' Administration Lung Cancer Study Group.

Patients underwent a clinical evaluation with full blood count, biochemistry and chest radiography on day 1 of each cycle. All other imaging, e.g. ultrasound or CT scan demonstrating metastatic disease, was repeated after three and six courses of cytotoxic therapy and if progressive disease was suspected. Patients who completed therapy were monitored every 3 months or more often as clinically indicated.

### EVALUATION OF RESPONSE AND TOXICITY

### Response

The efficacy of treatment was evaluated in terms of response, duration of response and survival. Response criteria were those defined by the WHO. Patients were assessable for response provided they completed one or more courses of treatment. Time to progression was calculated as the time from start of cytotoxic therapy until definite progression or death. Survival was measured from the first day of treatment to the date of death (of any cause) or last follow-up.

### Toxicity

The WHO criteria were used to report toxicity. All patients who received at least one dose were considered evaluable for toxicity. The toxicity is reported as the worst experienced for all cycles per patient.

**Table 1** Characteristics of 70 patients with SCLC treated with epirubicin and etoposide

| | |
|---|---|
| Number of patients | 70 |
| Male | 42 |
| Female | 28 |
| Age (years) | |
| Median | 64 |
| Range | 39–84 |
| Performance status (WHO) | |
| 0 | 13 |
| 1 | 40 |
| 2 | 13 |
| 3 | 4 |
| Limited disease | 35 |
| Extensive disease | 35 |
| Sites of metastases | |
| Liver | 22 |
| Bone | 16 |
| Brain | 3 |
| Adrenal | 2 |
| Cutaneous | 1 |
| other | 5 |
| ≥ 2 sites | 11 |
| Sodium ≤ 130 mmol $l^{-1}$ | 11 |
| SIADH | 3 |
| LDH | |
| > 200 iu $l^{-1}$ | 29 |
| > 1000 iu $l^{-1}$ | 4 |

### Symptomatic improvement

All patients were asked to assess the severity of their symptoms, e.g. cough, haemoptysis, dyspnoea and chest pain, on a five-point scale (0–4) on the first day of treatment for each cycle. Any reduction in severity by ≥ 1 on this scale was taken as evidence of symptomatic improvement.

*Statistical analysis*

Survival and duration of response curves were estimated by the Kaplan–Meier method (Kaplan and Meier, 1958). In the survival analysis, all causes of death were considered. All median values are followed by range, and frequencies expressed in percentages followed by 95% confidence intervals (CIs).

### RESULTS

### Response to treatment and survival

Patient characteristics are shown in Table 1. Median age was 64 years (range 39–84) and median performance status (PS) (WHO) was 1 (range 0–3). Seventeen (24.2%) patients had a PS ≥ 2, and a total of 40 (57.1%) patients were hyponatraemic and/or had a raised lactate dehydrogenase (LDH). Thus, a total of 13 (37.1%) patients with limited disease and 20 (57.1%) patients with extensive disease had poor prognostic features.

Of the 70 patients treated with the EE combination, 59 were evaluable for response. Eleven were not assessable for response to chemotherapy for the following reasons: five patients died before the second cycle of chemotherapy; two died of neutropenic sepsis; and three suffered unexplained deaths. Two patients developed

© Cancer Research Campaign 1997

prolonged infective complications after recovery from neutropenia in the first cycle precluding further chemotherapy. Of the remaining excluded patients, two had fatal gastrointestinal bleeds, one had a cerebrovascular event and one was lost to follow-up. Two patients progressed after the first treatment and are included in the analysis.

The overall response rate was 64.4% (CI 52–76.8%). Fourteen patients (23.7%, CI 12.7–34.7%) had a complete remission, 24 (40.7%, CI 28–53.4%) a partial remission, eight stable disease and 13 progressed on treatment. In patients with limited disease, 24 had an objective response (77.4%, CI 62.4–92.4%), including ten (32.25%, CI 15.46–49.04%) complete responses. Four (14.2%, CI 1.1–27.3%) patients with extensive disease had a complete response.

Median time to progression for all responders was 7 months (range 1–70 +). The median time to progression was 8 months for patients with limited disease (range 1–70 +) and 5.75 months (range 1–16) for patients with extensive disease. The median survival was 10.5 months (range 0.5–70 +) in patients with limited disease and 7 months (range 0.5–24) for those with extensive disease. There was a statistically significant difference in survival between patients with limited disease and those with metastatic disease ($P = 0.02$).

Eleven patients (31.4%) with limited disease survived 12 months or more, and two (5.7%) survived more than 24 months, with one long-term ongoing survivor at 70 months. Only six patients (17.1%) with extensive disease survived 12 months or more.

### Symptomatic improvement

An improvement of symptoms occurred in 79% (CI 68.3–89.7%) of patients with shortness of breath, 80% (CI 69.5–90.5%) with cough, 81% (CI 71.5–92.1%) with haemoptysis and 68% (CI 55.7–80.3%) with pain. In a total of 19 of the 40 patients who presented with weight loss, an increase in body weight was noted at completion of treatment.

### Toxicity

Eleven patients were hospitalized for neutropenic sepsis. WHO grade 3 and 4 haematological toxicity was as follows: neutropenia was observed in 13 (18.5%) patients; thrombocytopenia requiring platelet transfusion in two (2.8%) patients; and anaemia in three (4.2%) patients.

Two toxic deaths were documented. Both patients died with sepsis while neutropenic.

A 25% dose reduction of both epirubicin and etoposide was necessary in 18 patients. This included ten patients with neutropenic infection, two patients with thrombocytopenia, four patients with mucositis, one patient with diarrhoea and one patient because of age. Three patients had a 25% dose reduction of epirubicin alone; two with abnormal liver function tests but normal bilirubin and one with ischaemic heart disease.

Chemotherapy was delayed in three patients because of low blood count; in one patient because of diarrhoea and in eight patients to allow recovery from neutropenic infection.

Alopecia was almost universal. Grade 3 or 4 mucositis, anorexia, nausea and vomiting and diarrhoea was seen in 21.4%, 11.4%, 8.5% and 5.7% patients respectively.

### Radiotherapy

Eight patients with limited disease who responded to chemotherapy received thoracic irradiation whereas 16 did not. Radiotherapy to the chest was also given to four patients as palliation and to four at the time of relapse. Cranial irradiation was given for palliation of cerebral metastases in 13 patients.

### DISCUSSION

Although SCLC is highly sensitive to both chemotherapy and radiotherapy, the majority of patients are destined to die of progressive disease. Many different chemotherapy regimens have been evaluated but no major advances have resulted from autologous bone marrow transplantation, the use of granulocyte colony-stimulating factor (G-CSF) to escalate the doses or to increase the dose frequency, or by novel combinations of existing drugs (Klastersky, 1995). Until such time as new drugs and drug combinations have been identified that enhance both response rates and survival durations, treatment should be with a regimen that combines efficacy with patient acceptability, low toxicity and cost.

The combination of epirubicin and etoposide has been tested in several phase II trials but always in addition to other drugs, such as cyclophosphamide (Macchiarini et al, 1990*b*), cisplatin (Rosell et al, 1992), ifosfamide (Sculier et al, 1995) or in four drug regimens (Bamberga et al, 1992).

Variable doses of anthracycline, either doxorubicin (45 mg $m^{-2}$) or epirubicin (60 mg $m^{-2}$ or 90 mg $m^{-2}$), given on day 1 with ifosfamide (1.5 g $m^{-2}$) and etoposide (80 mg $m^{-2}$) day 1–3, resulted in response rates varying from 73% to 83% (Sculier et al, 1995). The overall median survival was 42 weeks, which is very similar to our own results. Higher doses of epirubicin (100–120 mg $m^{-2}$) given on day 1 with cisplatin (70 mg $m^{-2}$) and etoposide (60–80 mg $m^{-2}$) for days 1–5 had a higher response rate at 81% and median survival of 13 months (Rosell et al, 1992). However, this was at the expense of increased toxicity with 53% neutropenic fever and 40% stomatitis compared with 15% grade 3 and 4 infection and 3% stomatitis in the ifosfamide-containing combination. Our own toxicity data showed the two-drug regimen to have a 15.7% rate of neutropenic sepsis and 21.4% for stomatitis. The planned epirubicin dose intensity up to 120 mg $m^{-2}$, which was approximately 30% higher than that used by both Sculier and ourselves, may well explain both the increased activity seen with this regimen as well as the marked toxicity.

Unfortunately, even though both epirubicin and etoposide are active single agents for SCLC, our response and survival data may be compromised as many of our patients had poor prognostic features such as hyponatraemia (11 patients), elevated LDH (29 patients) and syndrome of inappropriate antidiuretic hormone (three patients). Poor performance status (PS 3) patients were also included and not all the patients with limited disease received thoracic irradiation, which has subsequently been demonstrated to add to the benefit of chemotherapy. The value of thoracic irradiation in patients with limited disease was uncertain at the time this protocol was initiated.

The addition of a third drug or combining etoposide with a platinum compound might have been beneficial. The combination of carboplatin with oral etoposide gave an overall response rate of 67%, with a complete response of 21% in 106 patients (Pfeiffer et al, 1995). These results are similar to our own (overall response rate 66.6% and complete response 24.5%). Their response rate in patients with limited disease was higher (89% with CR 41%) than ours (77.4% with CR 32%) and the median survival at 15 months was comparable to that obtained in trials in which both the carboplatin and etoposide were administered intravenously.

© Cancer Research Campaign 1997

Myelosuppression was the main toxicity, with grade 3 or 4 leucopenia observed in 20% of patients and thrombocytopenia in 16% of patients. In a further study, a combination of oral etoposide 100 mg $m^{-2}$ for 7 days and carboplatin 150 mg $m^{-2}$ i.v. on day 1 was given to 47 elderly (> 45 years) or medically unfit patients with SCLC (Evans et al, 1995). Of the 38 patients evaluable for response, 71% responded with a complete response in 29% of patients. The median survival was 46 weeks and the treatment was generally well tolerated, with the major toxicity being neutropenia. Both these studies and our study show that oral etoposide can be incorporated in regimens for SCLC administered on an outpatient basis that are effective and well tolerated.

The relatively poor results in terms of survival in our study would imply that our regimen has less activity than a platinum–etoposide combination. It is not clear why this should be. It is perhaps accentuated by reporting the results of unselected patients compared with highly selected patients in phase I/II studies. Interestingly, a very short median survival of only 20 weeks was seen in an MRC study comparing ECMV (etoposide, cyclophosphamide, methotrexate and vincristine) with EV (etoposide and vincristine) (Bleehen et al, 1996). This was in part thought to be related to patients of poor performance status as well as a high proportion (17%) of early deaths, which may have been due to undiagnosed sepsis. Our own early death rate was lower at 10%, but was still a significant proportion. In addition, the efficacy of oral etoposide as a single agent has recently been questioned (Thatcher et al, 1996; MRC Lung Cancer Working Party, 1996). A multicentre randomized trial of oral etoposide compared with standard intravenous multidrug chemotherapy was stopped owing to poor response rates of 45% vs 51% respectively.

In view of the poor duration of survival we reported with oral etoposide and epirubicin, we have decided to consider a platinum-based regimen in future.

### REFERENCES

Abratt RP, Willcox PA and Hewitson RH (1987) Etoposide combination therapy for small cell carcinoma of the lung. *Cancer Chemother Pharmacol* **20**: 83–84

Albain KS, Crowley JJ, Le Blanc M and Livingston RB (1990) Determinants of improved outcome in small cell lung cancer: An analysis of the 2,580 patient Southwest Oncology Group database. *J Clin Oncol* **8**: 1563–1574

Bamberga M, Michetti G, Bamberga P, Ori-Belometti M, Gritti G, Sarti E and Villa R (1992) Cyclophosphamide, epirubicin, etoposide, cisplatinum (CEVP) in combination with radiotherapy: evaluation of a protocol adopted for 6 years in 148 cases of small cell lung cancer. *Tumori* **78**(5): 333–337

Blackstein M, Eisenhauer EA, Wierzbicki R and Yoshida S (1990) Epirubicin in extensive small cell lung cancer: A phase II study in previously untreated patients. A National Cancer Institute of Canada Clinical Trials Group study. *J Clin Oncol* **8**: 385–389

Bleehen NM, Girling DJ, Hopwood P, Lallemand G, Machin D, Stephens RJ and Bailey AJ. MRC Lung Cancer Working Party. (1996) Randomised trial of four-drug vs less intensive two-drug chemotherapy in the palliative treatment of patients with small-cell lung cancer (SCLC) and poor prognosis. *Br J Cancer* **73**: 406–413

Eckhardt S, Kolaric K, Vukas D, Kanitz E, Schoket Z, Jassem J, Vuletic L, Jelic S, Mechl Z, Koza I et al (1990) Phase II study of 4-epi-doxorubicin in patients with untreated extensive small cell lung cancer. *Med Oncol Tumor Pharmacother* **7**(1): 19–23

Evans WK, Radwi A, Tomiak E, Logan DM, Martins H, Stewart DJ, Goss G, Maroun JA and Dahrouge S (1995) Oral etoposide and carboplatin: effective therapy for elderly patients with small cell lung cancer. *Am J Clin Oncol* **18**(2): 149–155

Hansen HH (1992) Management of small cell cancer of the lung. *Lancet* **339**: 846–849

Johnson DH, Kim K, Turrist AT, Sause W, Komaki R, Wagner H and Blum R (1994) Cisplatin and etoposide plus concurrent thoracic radiotherapy administered once versus twice daily for limited-stage small cell lung cancer (abstract). *Proc Am Soc Clin Oncol* **13**: 333

Kaplan EL and Meier P (1958) Nonparometric estimation from incomplete observations. *J Am Stat Assoc* **53**: 457–481

Klastersky J (1995) Small cell lung cancer: Can treatment results be improved further? *Semin Oncol* **22** (suppl. 2): 1–2

Loehrer PJ Sr (1995) Palliative therapy: Extensive small cell lung cancer. *Semin Oncol* **22** (2 suppl. 3): 40–44

Loehrer PJ SR, Ansari R, Gonin R, Monaco F, Fisher W, Sandler A and Einhorn LH (1995) Cisplatin plus etoposide with and without ifosfamide in extensive small-cell lung cancer: A Hoosier Oncology Group Study. *J Clin Oncol* **13**: 2594–2599

Macchiarini P, Danesi R, Mariotti R, Marchetti A, Fazzi P, Bevilacqua G, Mariani M, Giuntini C, Del Tacca M and Angeletti CA (1990*a*) Phase II study of high-dose epirubicin in untreated patients with small cell lung cancer. *Am J Clin Oncol* **13**(4): 302–307

Macchiarini P, Chella A, Riva A, Mengozzi G, Silvano G, Solfanelli S and Angelleti CA (1990*b*) Phase II feasibility study of high dose epirubicin-based regimens for untreated patients with small cell lung cancer. *Am J Clin Oncol* **13**: 495–500

Pedersen AG and Hansen HH (1983) Etoposide (VP-16) in the treatment of lung cancer. *Cancer Treat Rev* **10**: 245–264

Pfeiffer P, Sorensen P and Rose C (1995) Is carboplatin and oral etoposide an effective and feasible regime in patients with small cell lung cancer? *Eur J Cancer* **31A**: 64–69

Plosker GL and Faulds D (1993) Epirubicin: A review of its pharmacodynamic and pharmacokinetic properties, and therapeutic use in cancer chemotherapy. *Drugs* **45**: 788–856

Rosell R, Carles J, Abad A, Jimeno JM, Moreno I, Barnada A, Ribelles N and Haboubi N (1992) Phase II feasibility study of high dose epirubicin plus etoposide and cisplatin (HDEEC) regimen in small cell lung cancer. *Investigational New Drugs* **10**(2): 123–128

Sculier JP, Bureau G, Giner V, Thiriaux J, Michel J, Berchier MC, Van Cutsem O, Kustner U, Kroll F, Mommen P, Paesmans M and Klastersky J (1995) Induction chemotherapy with ifosfamide, etoposide and anthracycline for small cell lung cancer: experience of the European Lung Cancer Working Party. *Semin Oncol* **22** (1 suppl. 2): 18–22

Slevin ML, Clark PI, Joel SP, Malik S, Osborne RJ, Gregory WM, Lowe DG, Reznek RH and Wrigley PF (1989) A randomised trial to evaluate the effect of schedule on the activity of etoposide in small cell lung cancer. *J Clin Oncol* **7**: 1333–1340

Thatcher N, Clark PI, Girling DJ, Hopwood P, Twiddy S, Stephens RJ, Baily AJ and Machin D. MRC Lung Cancer Working Party (1996) Comparison of oral etoposide and standard intravenous multidrug chemotherapy for small-cell lung cancer: a stopped multicentre randomised trial. *Lancet* **348**: 563–566

Wolff AC, Ettinger DS, Neuberg D, Comis RL, Ruchdeschel JC, Bonomi PD and Johnson DH (1995) A phase II study of Ifosfamide, carboplatin and oral etoposide chemotherapy for extensive disease small cell lung cancer: An Eastern Cooperative Oncology Group pilot study. *J Clin Oncol* **13**: 1615–1622

© Cancer Research Campaign 1997